# Irritable Bowel Syndrome: A Hallmark of Psychological Distress in Women?

**DOI:** 10.3390/life15020277

**Published:** 2025-02-11

**Authors:** Giuseppe Marano, Gianandrea Traversi, Roberto Pola, Antonio Gasbarrini, Eleonora Gaetani, Marianna Mazza

**Affiliations:** 1 Unit of Psychiatry, Fondazione Policlinico Universitario A. Gemelli IRCCS, 00168 Rome, Italy; mariannamazza@hotmail.com; 2 Department of Neurosciences, Università Cattolica del Sacro Cuore, 00168 Rome, Italy; 3 Unit of Medical Genetics, Department of Laboratory Medicine, Ospedale Isola Tiberina-Gemelli Isola, 00186 Rome, Italy; 4 Section of Internal Medicine and Thromboembolic Diseases, Department of Internal Medicine, Fondazione Policlinico Universitario A. Gemelli IRCCS, Università Cattolica del Sacro Cuore, 00168 Rome, Italy; roberto.pola@policlinicogemelli.it; 5 Department of Medical and Surgical Sciences, Fondazione Policlinico Universitario A. Gemelli IRCCS, Università Cattolica del Sacro Cuore, 00168 Rome, Italy; 6 Department of Translational Medicine and Surgery, Fondazione Policlinico Universitario A. Gemelli IRCCS, Università Cattolica del Sacro Cuore, 00168 Rome, Italy; 7 Unit of Internal Medicine, Cristo Re Hospital, 00167 Rome, Italy

**Keywords:** irritable bowel syndrome, psychological distress, women, anxiety, depression

## Abstract

Irritable bowel syndrome (IBS) is a functional gastrointestinal disorder characterized by abdominal pain, bloating, and altered bowel habits. Women are disproportionately affected by IBS due to a complex interplay between genetic, environmental, and psychosocial factors, along with a crucial role of the gut–brain axis in modulating both bowel function and pain perception. Evidence suggests a strong association between psychological distress and IBS symptoms. Women with IBS report higher levels of psychological distress compared to men, and sex is a biological variable that shapes several aspects of the mechanisms, epidemiology, and clinical manifestations of IBS. This paper explores the bidirectional relationship between psychological factors and IBS with a focus on women. Stress, anxiety, depression, and childhood trauma contribute to IBS symptomatology, and societal and biological factors unique to women may exacerbate this condition. Strategies for integrated care approaches and gender-specific treatment strategies to improve patient outcomes and quality of life are needed.

## 1. Introduction

Irritable bowel syndrome (IBS) is a prevalent condition affecting 10–20% of the global population. It is considered a common functional gastrointestinal disorder characterized by chronic or recurrent abdominal pain associated with altered bowel habits, such as diarrhea (IBS-D), constipation (IBS-C), or a mix of both (IBS-M) [1]. Unlike structural gastrointestinal disorders, IBS lacks detectable anatomical abnormalities or inflammatory markers, leading to its classification as a disorder of gut–brain interaction. The characteristic presentation of IBS is the presence of abdominal pain, typically relieved or exacerbated by defecation, alongside irregular bowel patterns. The Rome IV criteria, a standardized diagnostic framework, are widely used to identify IBS based on symptomatology [2]. IBS is strongly linked to disturbances in the gut–brain axis, which encompasses bidirectional communication between the central and enteric nervous systems, modulated by microbial, hormonal, and immune pathways [3]. Enhanced sensitivity to gastrointestinal stimuli, referred to as visceral hypersensitivity, is frequently observed in patients with IBS and contributes to symptom severity [4]. Dysbiosis, or an imbalance in gut microbial composition, is implicated in the pathogenesis of IBS. Alterations in microbiota can influence intestinal permeability, immune activation, and neuroendocrine signaling [5].

Women are 1.5 to 3 times more susceptible than men to IBS, suggesting a potential link between gender-specific factors and the disorder [6]. The mechanisms of irritable bowel syndrome (IBS) involve both central and peripheral mechanisms that alter several bowel functions. These dysfunctions are associated with motor, sensory, immune, barrier, and intraluminal perturbations [7]. While there is evidence that these mechanisms are altered in both females and males, sex represents a biological variable that impacts a number of aspects of the mechanisms, epidemiology, manifestations, and exacerbation of IBS [8].

The fact that women exhibit a higher prevalence of IBS compared to men is documented by a female-to-male ratio of approximately 2:1 [6]. This sex disparity extends to symptom severity, with women often reporting more intense abdominal pain, bloating, and diarrhea. Furthermore, women with IBS are more likely to experience a higher burden of psychological comorbidities, including anxiety and depression. Several factors may contribute to these sex-based differences. Hormonal fluctuations, particularly variations in estrogen and progesterone levels, may influence gut motility, visceral sensitivity, and immune function in women. Sex-specific differences in gut microbiota composition and function may also play a crucial role. Additionally, women may experience higher levels of stress and anxiety, which can exacerbate IBS symptoms through complex neuroendocrine and immune pathways [7,8].

The disorder has a significant impact on quality of life, often leading to psychological distress, reduced productivity, and increased healthcare utilization [9]. Workers with IBS report greater occupational stressors and work productivity impairments and suffer from psychological distress, a low quality of life, and medical and economic problems [10].

Research indicates that women with IBS report higher levels of psychological distress compared to their male counterparts [11]. This gender disparity in IBS and psychological distress may be attributed to various factors, including hormonal fluctuations, social and cultural stressors, and gender-specific coping mechanisms.

A systematic review examined the prevalence of anxiety and depression in patients with IBS and found that these psychological conditions are significantly more common in patients with IBS than in the general population [12]. Stress can exacerbate IBS symptoms, leading to a vicious cycle of worsening gastrointestinal and psychological symptoms. Additionally, the presence of PD in patients with IBS has been linked to poorer treatment outcomes and a reduced quality of life [12].

A genetic predisposition is recognized in IBS, and a heritable component of IBS has been demonstrated in twin and family studies, but whether these genetic factors differently predispose individuals to various IBS forms and/or subtypes remains unclear. It has been hypothesized that IBS is a multifactorial, polygenic complex genetic disorder, and a combination of genetic factors and environmental factors lead to alterations in gastrointestinal sensation and motor function that ultimately result in symptom manifestation [13]. A study identified and confirmed six genetic susceptibility loci for IBS. Implicated genes included NCAM1, CADM2, PHF2/FAM120A, DOCK9, CKAP2/TPTE2P3, and BAG6. The first four are associated with mood and anxiety disorders, or are expressed in the nervous system, or both [14]. A study associated variants at the locus 9q31.2 with the risk of IBS in women. Since this region has been linked to a series of human conditions that are under the influence of hormonal stimuli, especially those involving the mechanisms of action of sex hormones, authors speculate that this result may provide additional rationale for investigating the role of sex hormones and autonomic dysfunction in IBS [15].

**Figure 1:**
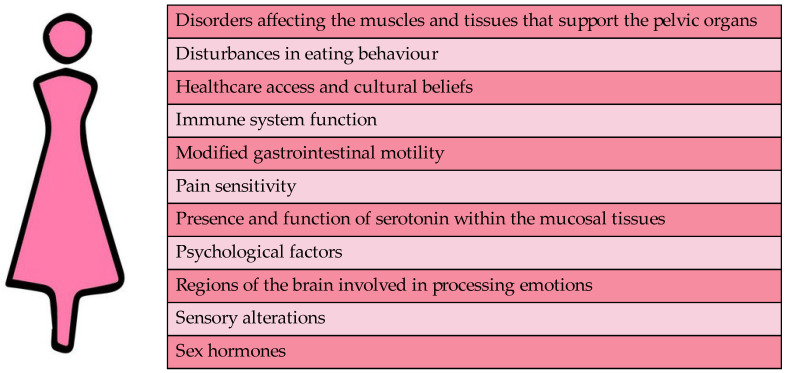
Sex differences in IBS: key contributing factors. This figure highlights the key factors contributing to the observed sex differences in IBS. It depicts the influences of hormones, immune function, psychosocial factors, and differences in gut physiology.

Figure 1 presents a comprehensive overview of the multifaceted factors that may contribute to this gender disparity. As illustrated in the figure, hormonal fluctuations and altered gut–brain interactions are among the key biological factors that may play a role. Additionally, psychological factors such as stress and anxiety, as well as social factors such as healthcare access and cultural beliefs, may also contribute to the higher prevalence of IBS in women. Understanding the complex interplay of these factors is essential for developing effective prevention and treatment strategies for IBS in women.

This paper reviews current research to investigate whether IBS may serve as a hallmark of psychological distress in women. A comprehensive literature search was conducted in the PubMed, CINAHL, EMBASE, and PsycINFO databases up to December 2024. The following keywords were utilized: “irritable bowel syndrome”, “gastrointestinal symptoms”, “psychological distress”, “women”, “anxiety”, and “depression”. To ensure the relevance and quality of the included studies, specific inclusion and exclusion criteria were applied. Studies were included if they explored the psychological aspects of IBS regarding women’s mental health. Clinical trials, observational studies, and systematic reviews were prioritized, while non-peer-reviewed articles, conference abstracts, case reports, and single-patient studies were excluded. Only peer-reviewed articles written in English were considered. Studies that were not published in English or that focused on pediatric populations were excluded from the research. Additional articles were identified through a manual search of the reference lists of the retrieved publications.

## 2. Pathophysiology of IBS

The pathophysiology of IBS is multifactorial and involves a complex interplay of various mechanisms. The key factors include visceral hypersensitivity, abnormal gut motility, and dysfunction of the autonomic nervous system. These mechanisms make the bowel susceptible to both exogenous and endogenous factors, such as gut flora alterations, psychosocial stressors, and dietary components [8].

IBS is classified as a functional disorder, meaning no identifiable structural abnormalities explain the symptoms. Central to its pathophysiology are disturbances in the gut–brain axis, a bidirectional communication system involving the central and enteric nervous systems. In particular, the pathophysiology of IBS can be considered multifactorial and is still incompletely understood, implicating neuroenteric communication through dysregulated signaling within the gut–brain axis, involving neurotransmitters like serotonin [16] and immune activation, and through low-grade inflammation and immune dysregulation, noted in subsets of patients with IBS, particularly following infections [17].

Among the key players in these processes are ion channels, which are integral membrane proteins regulating ion flow across cellular membranes. The role of these channels is increasingly recognized in the pathogenesis of IBS. Ion channels regulate diverse physiological processes, such as neurotransmission, muscle contraction, and secretion in the gastrointestinal tract. These channels are expressed in enteric neurons, smooth muscle cells, and epithelial cells. The dysfunction of ion channels disrupts homeostasis, contributing to symptoms of IBS. The transient receptor potential (TRP) channel family consists of nonselective cation channels activated by a variety of stimuli, including mechanical stress, temperature, and chemical signals. Several members of this family have been involved in IBS. TRPV1 is a heat-sensitive channel activated by capsaicin and protons. The upregulation of TRPV1 in colonic afferent nerves has been observed in patients with IBS, correlating with enhanced visceral hypersensitivity. Its activation contributes to abdominal pain by lowering the pain threshold in the gut [18]. TRPA1 is activated by irritants and oxidative stress and is linked to neurogenic inflammation and visceral hypersensitivity in IBS. Studies indicate that TRPA1 activation results in the release of pro-inflammatory neuropeptides, exacerbating pain perception [19]. TRPV4 plays a role in mechanosensation. The dysregulation of TRPV4 has been implicated in visceral hypersensitivity seen in IBS, particularly in pain exacerbated by colonic distension [20]. Voltage-gated ion channels contribute to the excitability of enteric neurons and muscle cells. Mutations in the SCN5A gene, encoding the Voltage-Gated Sodium Channel NaV1.5, are linked to IBS, especially in subtypes with constipation-predominant symptoms. These mutations affect the excitability of enteric neurons and GI motility [21]. Calcium channels modulate neurotransmitter release in the enteric nervous system. The dysregulation of these channels alters neuronal signaling, contributing to visceral hypersensitivity [22]. Chloride channels, such as CFTR (Cystic Fibrosis Transmembrane Conductance Regulator), regulate fluid secretion in the gut. Dysfunctional chloride transport can lead to diarrhea or constipation, common symptoms in IBS [23]. Mechanosensitive channels, including Piezo channels, detect mechanical stress in the gut. Abnormal activation of these channels may enhance pain sensitivity and alter motility, which are hallmark features of IBS [24].

Psychological factors seem to play a pivotal role: anxiety, depression, and stress are highly comorbid with IBS, exacerbating its clinical presentation and perpetuating gut–brain communication disturbances [25]. In particular, women with IBS often exhibit heightened visceral hypersensitivity, dysregulated gut motility, and altered microbiota composition, which may interact with psychological distress to perpetuate symptoms [16].

Recent studies have highlighted the role of immune activation in the development of IBS [26]. Autoantibodies targeting the enteric nervous system have been identified in some patients with IBS, suggesting an autoimmune component to the disorder [26]. Additionally, genetic factors and polymorphisms in human DNA may contribute to susceptibility to IBS [8]. Understanding these pathophysiological mechanisms is crucial for developing targeted treatments that address the underlying causes of IBS rather than just managing symptoms [27].

## 3. Gut–Brain Axis and IBS

**Figure 2:**
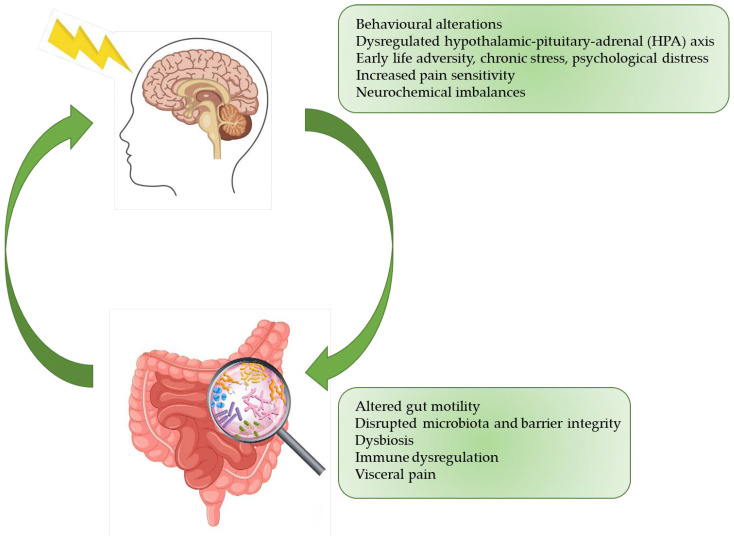
Bidirectional interactions between the brain and gut in IBS. This figure illustrates the complex bidirectional communication pathways between the brain and the gut in IBS. It emphasizes the role of this gut–brain axis in the development and maintenance of IBS symptoms.

The gut–brain axis plays a crucial role in the regulation of gastrointestinal functions. Chronic stress has been widely recognized as a trigger and exacerbating factor of irritable bowel syndrome. Figure 2 illustrates a conceptual framework outlining the potential mechanisms underlying the development of IBS. Adverse experiences during early life may predispose individuals to a dysregulated HPA axis response to subsequent stressors. Chronic activation of the HPA axis can lead to neurochemical imbalances, particularly in brain regions involved in pain processing, and consequently enhance pain sensitivity. Furthermore, bidirectional communication between the brain and gut can be disrupted, leading to alterations in gut motility, increased intestinal permeability, and dysbiosis. These gastrointestinal disturbances, in conjunction with central sensitization, contribute to the development and persistence of visceral hypersensitivity and IBS symptoms.

The gut–brain axis involves complex interactions between the gastrointestinal tract, central nervous system, autonomic nervous system, and immune signaling pathways. Dysregulation in this axis is thought to play a central role in the etiology of IBS. Neurotransmitters such as serotonin and dopamine, which influence both mood and gastrointestinal function, are frequently dysregulated in patients with IBS. Women with IBS demonstrate unique patterns of gut–brain communication, potentially linked to hormonal variations and psychosocial stressors [28].

Recent research has expanded the understanding of the gut–brain axis by highlighting the role of the vagus nerve, gut microbiota, and inflammatory mediators in IBS. The vagus nerve serves as a critical communication highway between the gut and brain, with alterations in vagal tone correlating with increased symptom severity and psychological distress [29]. Additionally, microbial metabolites such as short-chain fatty acids (SCFAs) influence neurochemical signaling and can modulate mood and gut motility, with disruptions in these pathways being linked to IBS [30].

Chronic low-grade inflammation has also emerged as a significant contributor to gut–brain axis dysfunction in IBS. Elevated levels of pro-inflammatory cytokines, such as interleukin-6 (IL-6) and tumor necrosis factor-alpha (TNF-α), have been observed in subsets of patients with IBS, suggesting an immune component to the disorder [31]. This inflammatory signaling can impact the integrity of the blood–brain barrier and lead to alterations in central nervous system functioning, further exacerbating symptoms [32].

Advances in neuroimaging have provided further insights into the gut–brain axis in IBS. Functional MRI studies have revealed altered connectivity in brain regions responsible for pain processing, emotional regulation, and interoception, such as the anterior cingulate cortex, amygdala, and insula. These findings underscore the importance of integrated approaches to address both central and peripheral mechanisms in IBS management [25].

## 4. Microbiota Alterations in IBS

The gut microbiota, a diverse ecosystem of microorganisms residing in the gastrointestinal tract, plays a critical role in maintaining gut health and modulating the gut–brain axis. Dysbiosis, or microbial imbalance, has been consistently observed in patients with IBS. Studies report reduced microbial diversity and alterations in the abundance of key bacterial species, such as reductions in *Lactobacillus* and *Bifidobacterium* populations and an increase in pathogenic strains like *Escherichia coli* [33].

Emerging research highlights the role of microbial metabolites, such as short-chain fatty acids (SCFAs) and tryptophan derivatives, in influencing gut motility, intestinal barrier integrity, and neurochemical signaling. SCFAs, produced through the fermentation of dietary fibers by gut bacteria, have anti-inflammatory properties and are critical for maintaining gut health. Disruptions in SCFA production have been implicated in the pathophysiology of IBS [30].

Recent advances in metagenomics and metabolomics have provided deeper insights into the functional alterations of the gut microbiome in IBS. For example, studies using 16S rRNA sequencing have identified specific microbial signatures associated with diarrhea-predominant (IBS-D) and constipation-predominant (IBS-C) subtypes. These findings suggest that tailored microbiome-targeted therapies can offer a promising avenue for treatment [34]. Probiotics and prebiotics have gained attention for their potential to restore microbial balance and alleviate IBS symptoms. Clinical trials have demonstrated that multi-strain probiotics, including *Lactobacillus* and *Bifidobacterium* species, can reduce bloating, abdominal pain, and stool irregularities [1]. However, the efficacy of these interventions varies, highlighting the need for personalized approaches based on individual microbiome profiles.

The gut virome and fungal communities are also gaining recognition as contributors to the pathogenesis of IBS. Preliminary studies suggest that bacteriophages and fungal species, such as *Candida*, may interact with bacterial populations to influence gut health and symptomatology. These findings underscore the complexity of the gut ecosystem and its relevance to IBS [35]. In addition, there are potentially distinct sex-related differences in the mucosal microbiomes of patients with IBS, supporting the importance of studying sex-specific mechanisms in the pathophysiology of IBS [36,37]. In women with IBS, lower abundances of *Coriobacteriaceae* and *Shuttleworthia* and increased abundances of *Bacteroidales*, *Christensenellaceae Christensenella*, *Anaerovorax*, *Mogibacterium*, and *Psuedobutyrivibrio* species have been observed compared to men with IBS [38].

Overall, microbiome research underscores the importance of gut microbial composition and functionality in IBS. Although there are clinical trials that have made good progress, more standardized, more generalized, larger-scale, and multi-omics clinical studies are missing. Future research integrating multi-omics approaches may unlock novel therapeutic targets, paving the way for microbiome-based precision medicine in IBS management.

## 5. The Role of Stress in IBS

Stress is a well-established trigger for IBS symptoms. The activation of the HPA axis during acute or chronic stress leads to elevated levels of cortisol and other stress mediators, which influence gut motility, permeability, and immune activation [39]. Women with IBS often report heightened symptom severity during stressful life events, such as bereavement, work challenges, or caregiving responsibilities [40]. Hormonal influences further compound the effects of stress in women. Fluctuations in estrogen and progesterone levels, particularly during the menstrual cycle, can modulate the stress response and exacerbate gastrointestinal symptoms. Significant associations between endometriosis and IBS, and a linear relationship between acyclic pelvic pain severity and the odds of IBS, have also been observed [41,42]. There is still debate about a possible association between polycystic ovary syndrome (POCS) and IBS; it is clear that several common potential pathways may directly and indirectly contribute to the interaction between IBS and PCOS, including an alteration in sex hormones or the gut–brain axis, the dysregulation of neurotransmitters and inflammatory factors, metabolic or reproductive disturbances, and psychological, environmental, and lifestyle factors [43].

Pregnant and postpartum patients with IBS have higher odds of psychological comorbidities in addition to medical comorbidities, such as migraines, connective tissue diseases, and autoimmune diseases [34].

Menopause—characterized by a decline in estrogen levels—can also lead to changes in gut function and symptom severity [44]. These hormonal dynamics underscore the importance of considering gender-specific factors in the management of IBS.

It has been outlined that women with IBS not only show greater psychological distress, but also lower body appreciation, higher body dissatisfaction, and higher self-criticism than controls. Body appreciation and self-criticism sequentially mediate the link between IBS status and both depression and anxiety. IBS is associated with reduced body appreciation, which in turn is linked to heightened self-criticism, thereby leading to elevated psychological distress. In other words, particularly in women, IBS negatively impacts body image appreciation, fostering self-critical judgments that exacerbate psychological symptoms [45].

The impact of disease on sexuality and intimacy is one of the main concerns of patients with IBD. The prevalence of sexual disfunction in patients with IBD has been reported to be 45–60% among women and 15–25% in men, with the most frequently associated factor being depression. Disease characteristics or disease activities are not associated with sexual dysfunction. Quality of sex life is a major component of quality of life in patients with IBD. In addition, in these patients, sexual dysfunction has a significant influence on the overall quality of life and is linked with poorer family functioning [46].

Additionally, societal pressures and higher rates of gender-based violence and discrimination may increase psychological distress in women, further aggravating IBS. Research has shown that women with IBS report higher levels of catastrophizing (a tendency to anticipate the worst outcomes) and reduced coping strategies compared to men [47]. Societal expectations and gender-specific stressors may contribute to the higher prevalence of IBS in women. Women are more likely to face caregiving responsibilities, workplace inequalities, and experiences of harassment or abuse. These stressors can increase their vulnerability to both psychological distress and IBS [6].

## 6. Psychological Distress and IBS

Psychological factors play a significant role in IBS. Studies revealed that up to 60% of patients with IBS also suffer from psychiatric comorbidities such as anxiety or depression [48]. Stress has been shown to exacerbate IBS symptoms by activating the hypothalamic–pituitary–adrenal (HPA) axis and increasing intestinal permeability. Chronic psychological distress can lead to the sustained activation of these pathways, contributing to the chronicity of IBS.

Previous studies have shown that IBS seems to be tightly linked to psychiatric disorders, more frequently anxiety disorders and depression. While psychiatric disorders are the most common comorbidity of IBS, it is often difficult to determine the temporal sequence of these conditions. It was found that major depressive disorder increases the risk of IBS, and IBS also increases the risk of MDD, demonstrating a significant bidirectional association between these conditions [49]. Psychiatric disorders and, in particular, mood disorders may play an important role in the development and persistence of IBS.

During pregnancy, maternal stress, signaled through elevated cortisol, can influence the fetus’s development and potentially the mother’s health. This interaction may have implications for the development of IBS and postpartum depression in the mother, and it may have potential health effects on the child. Maternal prenatal stress acts on the HPA axis, consequently increasing circulating cortisol levels, which in turn can affect the maternal gut microbiota. Maternal cortisol crosses the placenta, thus increasing cortisol-circulating levels in the fetus. This leads to the dysregulation of the HPA axis, conditioning the gut microbiota, microbial metabolites, and intestinal permeability in the fetus. Microbial metabolites, such as short-chain fatty acids (which also regulate the development of fetal enteric nervous system), can induce epigenetic changes and modulate a range of diseases. Implicit epigenetic stress information from the fetal enteric nervous system can be conveyed to the fetal central nervous system through the bidirectional microbiota–gut–brain axis, leading to perturbed functional connectivity among various brain networks and the dysregulation of affective and pain processes. This means that elevated fetal cortisol can influence HPA axis development in the fetus. This “programming” may predispose the child to stress-related disorders, including IBS and anxiety, later in life. On the other hand, elevated fetal cortisol may feed back into the maternal system, exacerbating the mother’s stress response and potentially contributing to IBS symptoms. In addition, the immune modulation required to maintain pregnancy may transiently suppress inflammation but can rebound postpartum, potentially exacerbating IBS symptoms in the mother [50,51].

Anxiety and depression are highly prevalent in IBS populations, with these psychiatric comorbidities amplifying symptom severity and reducing the quality of life [52]. These conditions contribute to heightened visceral hypersensitivity and dysregulation in brain–gut signaling. Studies suggest that patients with IBS in comorbidity with depression or anxiety present alterations in gut microbiota composition and cause immune, endocrine, and serotonergic system alterations relevant to the common pathophysiology of these comorbid conditions [53]. Neuroimaging studies have shown abnormal activity in the anterior cingulate cortex, insula, and amygdala—regions critical for emotional regulation and pain processing—in patients with IBS, suggesting a neurobiological basis for these associations [16]. The anterior cingulate cortex, a region involved in pain processing, has been investigated regarding its role in the regulation of visceral sensitivity and mental disorders. A crucial involvement of gamma-aminobutyric acid-producing (GABAergic) neurons’ movement to the lateral hypothalamus has been found to modulate anxiety-like behaviors, intestinal motility alterations, and visceral hypersensitivity [54].

Women with IBS are disproportionately affected by these psychological factors. Studies have shown that women are more likely to report higher levels of anxiety and depression compared to men, potentially due to societal pressures, hormonal fluctuations, and a greater tendency to internalize stress [55]. This internalization can perpetuate the cycle of psychological distress and exacerbate IBS symptoms. Emerging evidence suggests that early-life stressors, such as childhood trauma or adverse experiences, may predispose individuals to IBS by altering brain–gut axis development and increasing vulnerability to stress-related disorders [56]. In addition, although the likelihood of developing IBS due to adverse childhood experiences is similar for women and men, the higher prevalence of adverse childhood experiences and anxiety in women may contribute to the female predominance of IBS [57].

In a recent study, it was shown that a significant increase in 5-HT expression due to neonatal maternal separation in rodent models leads to alterations in intestinal structure and function; inhibiting 5-HT reversed these observed effects. Excess 5-HT in mice with early-life stress increased intestinal neural network density and promoted excitatory motor neuron expression. In particular, 5-HT activated the Wnt signaling pathway (a family of proteins that play critical roles in embryonic development and adult tissue homeostasis) through the 5-HT4 receptor, promoting neurogenesis within the intestinal nervous system. These findings confirm the regulatory role of 5-HT in the enteric nervous system and contribute to providing potential insights for the development of novel therapies for gastrointestinal disorders [58]. Interestingly, maternal separation resulted in increased visceral hypersensitivity while showing a trend for a sex-dependent increase in negative valence behavior in adulthood. Four clusters representing distinct pathophysiological domains reminiscent of the behavioral consequences of early-life stress have been identified (resilient, pain, immobile, and comorbid). These results suggest that the gut microbiota in early life shows sex-dependent alterations in each cluster that precede particular behavioral phenotypes in adulthood and might demonstrate the fact that stress-induced gut microbiota alterations appear in early life and contribute to the sex-specific susceptibility to specific gut–brain phenotypes in adulthood [59].

Addressing these underlying factors through psychological therapies may provide long-term relief for affected individuals.

In summary, women with IBS often report higher levels of psychological distress compared to men with the same condition. This disparity arises from a complex interplay of biological, hormonal, psychosocial, and cultural factors, which can intensify the experience and perception of IBS symptoms in women. Fluctuations in estrogen and progesterone during the menstrual cycle can increase visceral sensitivity, alter gut motility, and exacerbate IBS symptoms. This can lead to stronger emotional and psychological responses to IBS symptoms in women. Hormonal differences may enhance the activity of the hypothalamic–pituitary–adrenal (HPA) axis in women, leading to greater reactivity to stress and subsequent psychological distress. Hormonal changes during perimenopause and menopause can amplify symptoms of anxiety and depression, which are often comorbid with IBS [60].

Studies suggest that women with IBS have greater visceral hypersensitivity compared to men, meaning they perceive gastrointestinal pain and discomfort more acutely. This heightened pain perception can contribute to higher levels of anxiety, depression, and psychological distress. This increased sensitivity may result from differences in the gut–brain axis, which governs the bidirectional communication between the gut and the central nervous system [61].

Women may face unique stressors, such as caregiving responsibilities, societal expectations, and gender-based inequalities. These chronic stressors can exacerbate psychological distress and IBS symptoms. Women are more likely than men to have experienced trauma, including sexual or emotional abuse. Such experiences are strongly associated with IBS and psychological conditions like PTSD, anxiety, and depression [62].

Cultural norms and taboos about discussing bowel habits may lead women to feel embarrassed or isolated, increasing their psychological distress. Societal expectations for women to maintain a certain image or perform caregiving roles may make it harder for women to prioritize their health, exacerbating stress and frustration. In addition, women are more likely to internalize stress and emotions, which can worsen the psychological impact of chronic illnesses like IBS. Women with IBS are more likely to experience comorbid anxiety, depression, and somatization disorders than men. Psychological factors, such as catastrophizing factors, are more commonly reported in women and are associated with increased IBS symptom severity and distress. All of these conditions can amplify the perception of IBS symptoms and lead to a more profound emotional toll [63].

There is often a tendency for the underdiagnosis or misdiagnosis of IBS in women. In fact, women’s symptoms are sometimes dismissed or attributed solely to psychological causes, leading to feelings of invalidation and frustration. In addition, women are more likely than men to have their IBS attributed to stress or emotional issues, potentially downplaying their physiological concerns. This imbalance can increase distress and hinder effective treatment [64].

## 7. Integrated Interventions for Psychological Distress in Patients with IBS

The effective management of IBS requires addressing both the gastrointestinal and psychological aspects of the condition.

Due to the limitations of conventional treatments, and considering that treatments are not devoid of side effects and may not be cost-effective, there is a growing interest in complementary and alternative medicine approaches for symptom management. Herbal remedies have been extensively utilized in managing IBS symptoms. Peppermint oil, known for its antispasmodic properties, has demonstrated efficacy in reducing abdominal pain and overall IBS symptoms. A systematic review concluded that soluble fiber, such as psyllium, can improve constipation and global IBS symptoms [65]. Probiotics, which help maintain gut flora balance, have shown promise in alleviating IBS symptoms. However, evidence supporting the use of specific strains is limited [66].

Traditional Chinese medicine, including acupuncture and herbal formulations, has been employed in treating IBS. While some studies suggest benefits, the overall quality of evidence is limited, necessitating further rigorous research [67]. Mind–body therapies are also gaining recognition in IBS management. Gut-directed hypnotherapy seems to be a beneficial therapeutic option for certain patients [68].

Psychological therapies have shown significant efficacy in mitigating the impact of psychological distress on IBS. Cognitive behavioral therapy (CBT) and gut-directed hypnotherapy have been particularly effective in breaking the cycle of stress and symptom exacerbation. These interventions target maladaptive thought patterns, stress responses, and emotional regulation, offering relief to patients with significant psychological comorbidities [69]. Mindfulness-based stress reduction and acceptance–commitment therapy also offer promising results, helping patients manage their emotional responses to symptoms and improving their overall well-being [70].

Physical exercise through the modulation of gut microbiota may alleviate IBS symptoms. In particular, aerobic exercise (running, swimming, and cycling) enhances the diversity and abundance of beneficial gut bacteria (such as Lactobacillus and Bifidobacterium). These bacteria produce short-chain fatty acids that possess anti-inflammatory properties and support gut barrier integrity. Studies on patients with IBS participating in aerobic exercise programs have demonstrated significant improvements in the composition and diversity of gut microbiota, together with an alleviation of symptoms like abdominal pain and bloating. Additionally, exercise seems to positively influence mental health by reducing stress and improving mood, which can further relieve IBS symptoms via the gut–brain axis [71,72].

Patients with IBS often attribute the onset or worsening of gastrointestinal symptoms to food intake. In a Swedish study conducted on a population of predominantly women with IBS, subjects who consumed less carbohydrates, coffee, and dairy products and more fats, lactose-free dairy products, and nuts and seeds were compared with controls. In patients, symptom severity and gastrointestinal-specific anxiety were associated with reduced energy and increased carbohydrate intake, lower diet diversity, and worse diet quality. Poor diet quality was associated with a younger age, more severe IBS symptoms, anxiety, and depression [73].

Dietary modifications, such as the low-FODMAP diet, are commonly recommended for IBS management. FODMAP (Fermentable Oligosaccharides, Disaccharides, Monosaccharides, and Polyols) is an acronym for a certain class of carbohydrates (fermentable short-chain carbohydrates) which are more difficult to digest. The low-FODMAP diet temporarily restricts these carbohydrates to relieve uncomfortable symptoms and give the digestive system a rest, helping restore a healthy balance of gut flora [74]. A gluten-free diet and the Mediterranean diet have also demonstrated efficacy in improving IBS symptoms. It is important to carefully consider age or gastrointestinal, oncological, or cardiovascular comorbidities when prescribing specific diets to patients with IBS [75].

The efficacy of acupuncture has recently been demonstrated as a non-drug alternative therapy for visceral hyperalgesia-induced IBS: acupuncture can block the excessive stimulation of abnormal pain signals in the brain and spinal cord; it can also stimulate glial cells to block visceral hypersensitivity pain perception and cognition. Furthermore, acupuncture can regulate the emotional components of IBS by targeting hypothalamic–pituitary–adrenal axis-related hormones and neurotransmitters via relevant brain nuclei [76].

Pharmacological treatments include antispasmodics, probiotics, and serotonergic agents. The first-line treatment of constipation is the use of laxatives, with secretagogues being used when laxatives are ineffective. Anti-diarrheal drugs should be used as a first-line treatment for diarrhea, with second-line drugs including 5-hydroxytryptamine-3 antagonists, eluxadoline, or rifaximin, where available. The first-line treatment of abdominal pain should be the use of antispasmodics, with gut–brain neuromodulators being prescribed as a second-line treatment. Low-dose tricyclic antidepressants, such as amitriptyline, are preferred [77].

Women may benefit from tailored interventions that address hormonal influences on gut function [78]; for example, post-menopausal women with IBD who underwent hormone replacement therapy showed an improvement in their disease [79].

Vagus nerve stimulation may reduce constipation and abdominal pain and can open possibilities for responding to patient expectations [29,80].

Targeting ion channels offers promising therapeutic avenues for IBS. Antagonists of TRPV1 and TRPA1 have shown potential in reducing visceral pain. Similarly, modulators of voltage-gated sodium and calcium channels may help normalize neuronal excitability and motility. Emerging therapies aiming to correct chloride and mechanosensitive channel dysfunction are under investigation [81].

Some researchers have proposed a latent class analysis, a method of mathematical modeling, to show that patients with IBS can be classified into seven unique clusters based on a combination of gastrointestinal symptoms, abdominal pain, extraintestinal symptoms, and psychological comorbidity. These clusters can be used to predict the prognosis of IBS (e.g., symptom severity), healthcare use (e.g., consultation behavioral, prescriptions, and costs), and impact (e.g., quality of life, work and productivity, activities of daily living, and income). These clusters can also be used to increase the personalization of IBS treatment that better recognizes the heterogenous nature of the condition [9]. Of note, fecal microbiota transplantation holds promise as a microbiota-modulating treatment for major depressive disorder [82].

A multidisciplinary approach involving gastroenterologists, psychologists, dietitians, and gynecologists can provide comprehensive care for women with IBS. Addressing the interplay of psychological, hormonal, and gastrointestinal factors can lead to improved outcomes and quality of life.

## 8. Conclusions

Evidence underscores a significant interplay between psychological distress and IBS, particularly in women. While IBS is not solely a psychological condition, its strong association with stress, anxiety, and depression highlights the need for integrated care approaches. In women, the bidirectional relationship between psychological factors and IBS is shaped by hormonal fluctuations, heightened visceral sensitivity, and unique psychosocial stressors.

Recognizing IBS as a potential marker of psychological distress can help healthcare providers develop more effective, gender-specific treatment strategies to improve quality of life in women affected by this condition. Integrated care approaches should address the multifaceted nature of the condition and recognize the unique challenges faced by women. Including gynecologists can help address the interplay between IBS symptoms and hormonal changes. Psychological interventions, such as CBT and gut-directed hypnotherapy, can help manage psychological distress and reduce symptom severity. Integrating these therapies with pharmacological and dietary interventions—such as low-FODMAP diets and serotonergic agents, considering women’s specific nutritional needs, particularly during life stages like pregnancy or menopause—can provide a holistic approach to IBS management. For women whose IBS symptoms worsen during menstruation or menopause, hormonal therapy or oral contraceptives may help stabilize hormone levels and alleviate symptoms. Stress reduction techniques may help women manage physical and emotional stress, improving their overall well-being.

Integrated care includes trauma-informed approaches, recognizing that women with IBS may have a history of trauma, such as abuse, which influences their symptoms.

Gender-specific policies and resources ensure women have access to multidisciplinary care, flexible scheduling, and supportive services like childcare, which can reduce barriers to seeking treatment. For example, telemedicine options and online support groups also provide accessible platforms for ongoing care and peer support.

Healthcare providers trained in gender-sensitive communication are more likely to validate women’s experiences and avoid dismissing their symptoms as purely psychological. Building trust through empathetic care encourages women to share details about their symptoms and psychosocial stressors, leading to better diagnostic accuracy and treatment adherence.

Future research should continue to explore the interplay between psychological factors, gender-specific influences, and gastrointestinal symptoms to optimize treatment strategies.
